# Regulatory pathways and guidelines for nanotechnology-enabled health products: a comparative review of EU and US frameworks

**DOI:** 10.3389/fmed.2025.1544393

**Published:** 2025-03-05

**Authors:** Francisco D. Rodríguez-Gómez, Dominique Monferrer, Oriol Penon, Pilar Rivera-Gil

**Affiliations:** ^1^Asphalion SL, Barcelona, Spain; ^2^Integrative Biomedical Materials and Nanomedicine Lab, Department of Medicine and Life Sciences, Universitat Pompeu Fabra Barcelona Biomedicine Research Park (PRBB) Doctor Aiguader, Barcelona, Spain; ^3^OEM Technology Center, Werfen, Barcelona, Spain

**Keywords:** nanotechnology-enabled health products, regulatory pathways, European Union, United States, healthcare innovation, medical regulations

## Abstract

The integration of nanotechnology into healthcare has introduced Nanotechnology-Enabled Health Products (NHPs), promising revolutionary advancements in medical treatments and diagnostics. Despite their potential, the regulatory navigation for these products remains complex and often lagging, creating barriers to their clinical application. This review article focuses on dissecting the regulatory landscape for NHPs, particularly in the European Union and the United States, to identify applicable requirements and the main regulatory guidelines currently available for meeting regulatory expectations.

## 1 Introduction

### 1.1 Defining nanomedical technologies

Nanotechnology is an interdisciplinary field that encompasses the design, fabrication, and application of materials at the nanoscale, typically within the range of 1–100 nanometers. These materials, referred to as ‘nanomaterials’, possess unique properties due to their size, distinguishing them markedly from their larger, bulk material counterparts. Such properties are of significant interest for research and industrial applications, specifically in the field of health (1, 2).

**Figure 1:**
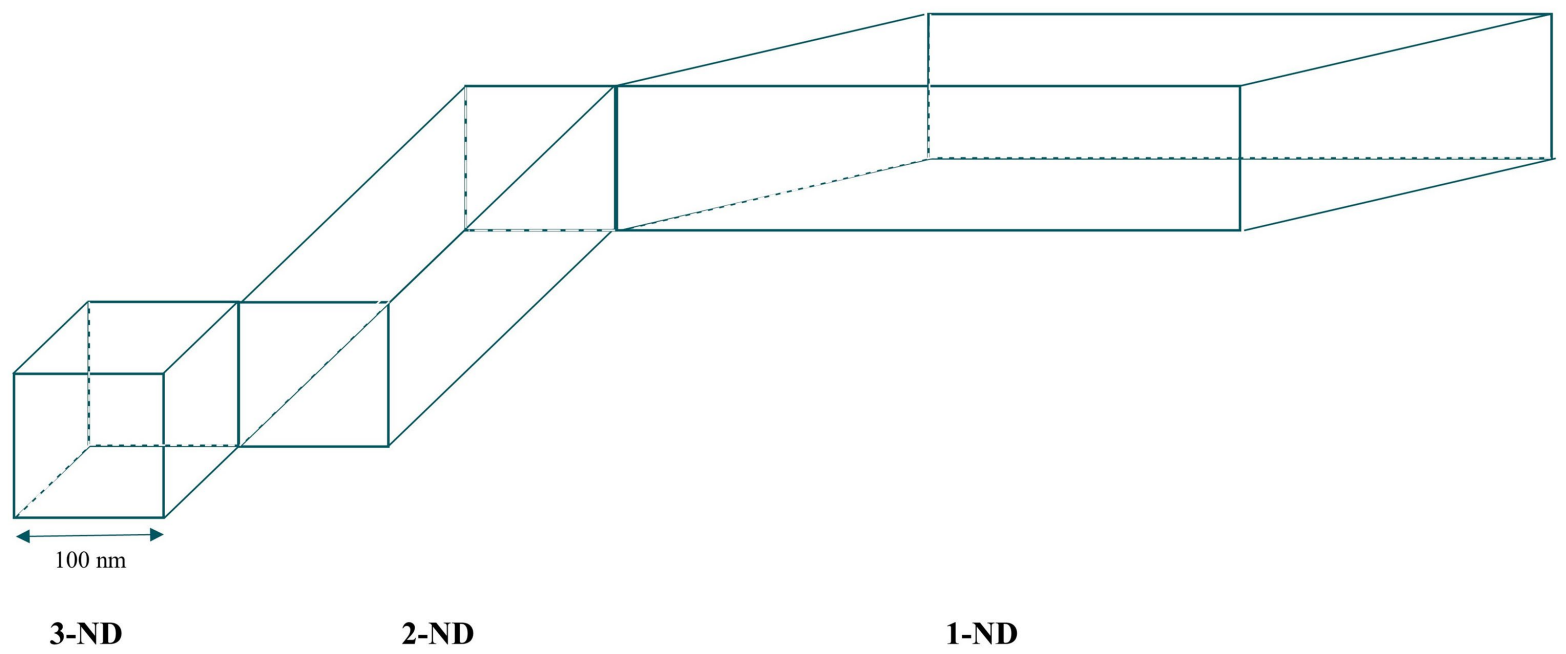
Types of nanomaterials based on the number of dimensions within the nanoscale.

Nanomaterials can be categorized based on their number of dimensions that fall within the nanoscale (3). This classification, depicted in Figure 1, includes:

- Three-dimensional nanomaterials (3-ND): all dimensions (x, y, z) are within the nanoscale. Examples include fullerenes, quantum dots, and nanoparticles.
- Two-dimensional nanomaterials (2-ND): only two dimensions fall within the nanoscale. This category comprises nanofibers, nanotubes, nanorods, and nanowires.
- One-dimensional nanomaterials (1-ND): characterized by a single dimension at the nanoscale, these are flat structures such as nanosheets, nanowalls, and nanolayers.

**Figure 2:**
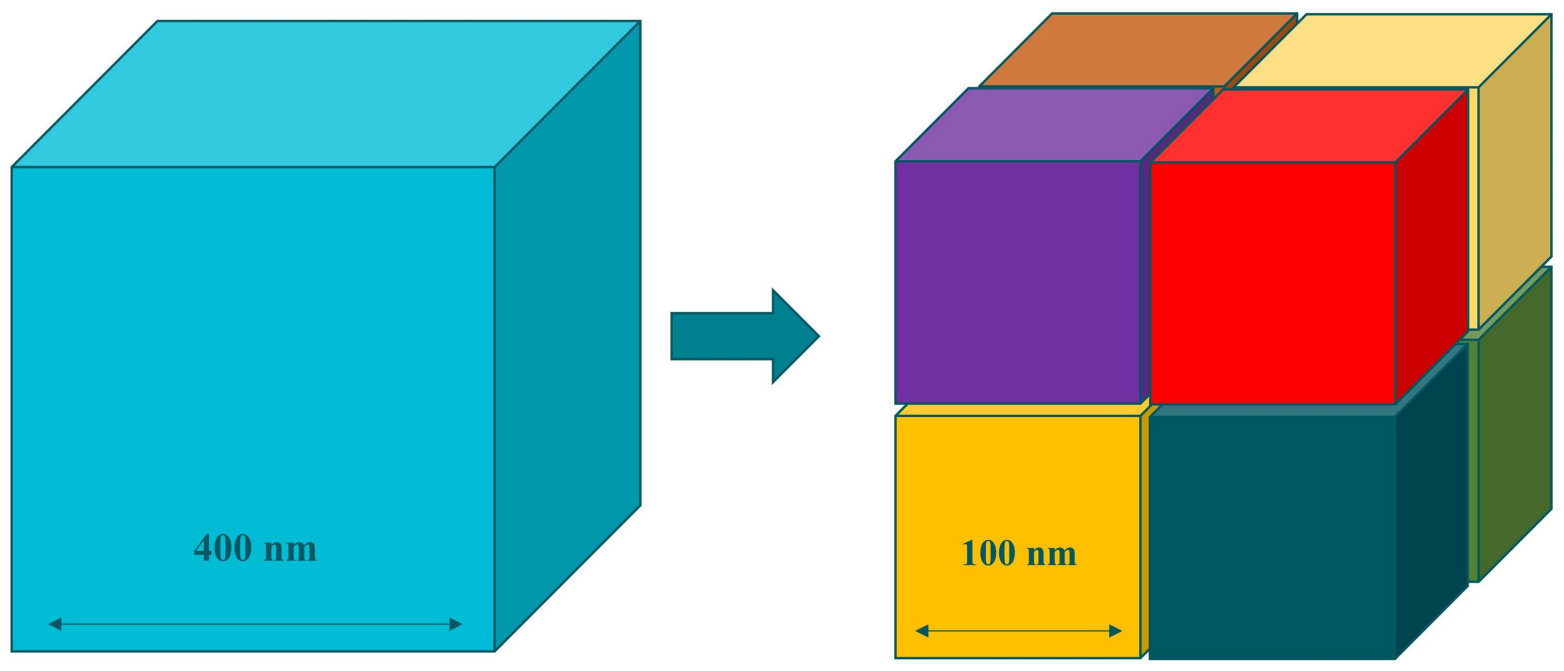
Increased surface area in nanomaterials.

The basis of these special properties of nanomaterials lies in their increased surface area. As bulk materials are subdivided into nanoscale entities, the total volume remains the same, yet the cumulative surface area of all entities is exponentially increased (Figure 2). This enhanced surface area increases the exposure to the outer media, which improves the reactivity of nanomaterials when compared to materials above the nanoscale (4).

Furthermore, the high surface-to-volume ratio in nanomaterials means that the majority of atoms are located at the surface. Forces applying to an atom are compensated except for those at the surface. The bond formation causes electrons to rearrange into lower energy levels as per Hund’s Rule of Maximum Multiplicity. In nanomaterials, with most atoms at the surface, there are fewer interatomic bonds, resulting in higher surface energy (5). This phenomenon also explains the reduction in the binding energy between atoms, which leads to a significant decrease in melting temperature for nanomaterials relative to their bulk material counterparts (6).

In addition to these distinct attributes, one of the key benefits of innovation in nanotechnology is the ability to fine-tune the physicochemical properties of materials, creating nanomaterials with tailored characteristics for a variety of applications. Specifically, the application of nanotechnology in the health sciences is broadly referred to as ‘nanomedicine’ or ‘nanopharmacy’ (7, 8). This discipline aims to develop tools, hereinafter referred to as nanotechnology-enabled health products (NHPs), for the diagnosis, prevention, and treatment of diseases, thereby revolutionising medical approaches and therapies (9–13).

### 1.2 Evolutionary overview of NHPs

The prefix ‘nano’ derives from the Ancient Greek ‘nanos’ (*νᾶνος*), meaning ‘dwarf’. It first emerged in the scientific lexicon in 1956 and gained formal recognition in 1960 during the General Conference on Weights and Measures (*Onzième Conférence Générale des Poids et Mesures*) (14). Richard Feynman, notable for his influential 1959 lecture, ‘*There’s plenty of room at the bottom*’, contributed to the conceptual understanding of manipulating matter at an atomic level, reflecting the collaborative spirit of scientific discovery in nanotechnology (15).

The field has been shaped by numerous scientists, with early surface phenomena research by Gauss, Young, Laplace, and Poisson. Wilhelm Ostwald in 1915 emphasized the significance of studying at smaller scales. Hermann Staudinger’s pioneering work in polymer chemistry in 1930 and Lev Landau’s DLVO theory in 1962 provided foundational knowledge about molecular interactions at the nanoscale. The invention of the scanning tunnelling microscope by Heinrich Rohrer and Gerd Binnig, who were awarded the Nobel Prize in Physics in 1986, enabled direct manipulation of atoms and molecules, significantly advancing the field of nanotechnology.

In the broader context of these developments, Norio Taniguchi, a Japanese scientist, introduced the term ‘nanotechnology’ in 1974. He described it as the precision engineering of materials at the atomic or molecular scale (16).

From this historical juncture, the fascination with nanomaterials and their myriad uses began to intensify. Despite being considered an emerging science, nanomedicine has been a subject of research since the 1990s, particularly its applications in medicine, medical technology, and pharmacology (17). The remarkable diagnostic and therapeutic capabilities of nanomaterials in healthcare are attributed to their size compatibility with cellular organelles, facilitating direct interactions with cells and thereby serving as potent agents for biological process modulation (18).

Early advancements in nanomedicine sought to enhance the pharmacokinetics of existing drugs. Nanomaterials as drug delivery vehicles enable controlled and tailored release, reducing dosages and minimizing adverse effects. They also offer favorable changes in clearance, retention, and half-life, improving therapeutic efficacy based on pharmacokinetic metrics such as area under the curve (AUC) and maximum concentration (C_max_) (19).

One of the earliest nanomaterial-based medicinal products to receive approval were the polyethylene glycol-coated nanoliposomal doxorubicin formulations, including Doxil® (approved by the FDA in 1995) and Caelyx® (approved by the EMA in 1996) (20). Currently, approximately seven hundred health-related products employ nanomaterials (21).

Nanomedicine reflects the broader evolution in medicine towards precision and personalized therapies (22). The field is at the forefront of employing detailed genetic profiling to devise customized diagnoses and treatments, accommodating the distinct genetic, phenotypic, and environmental factors influencing individual treatment responses. Recent efforts have seen the integration of nanotechnology with personalized medicine, bolstering drug delivery and molecular diagnostics. For instance, liposomal formulations enhance drug stability, reduce systemic toxicity, and enable targeted delivery in oncology applications (23). Polymeric nanoparticles can be engineered to carry therapeutic genetic material for precision-based interventions, thereby improving bioavailability while minimizing adverse effects. Nanomedicine also provides insights into individual genetic profiles, guiding the development of bespoke diagnostic and therapeutic strategies, including patient-tailored gene editing and biomarker-based diagnostics (24).

### 1.3 Nanomedicine in practice

Nanotechnology applications span a wide array of industries, including textiles, automotive, civil engineering, construction, solar technologies, environmental applications, transportation, agriculture, and food processing (25). This review, however, will concentrate on nanomaterial-based products that serve a medical purpose, i.e., NHPs. It is commonly held that NHPs have potential in four principal areas: nano-diagnosis, controlled drug delivery, treatment, and regenerative medicine (24, 26, 27).

**Figure 3:**
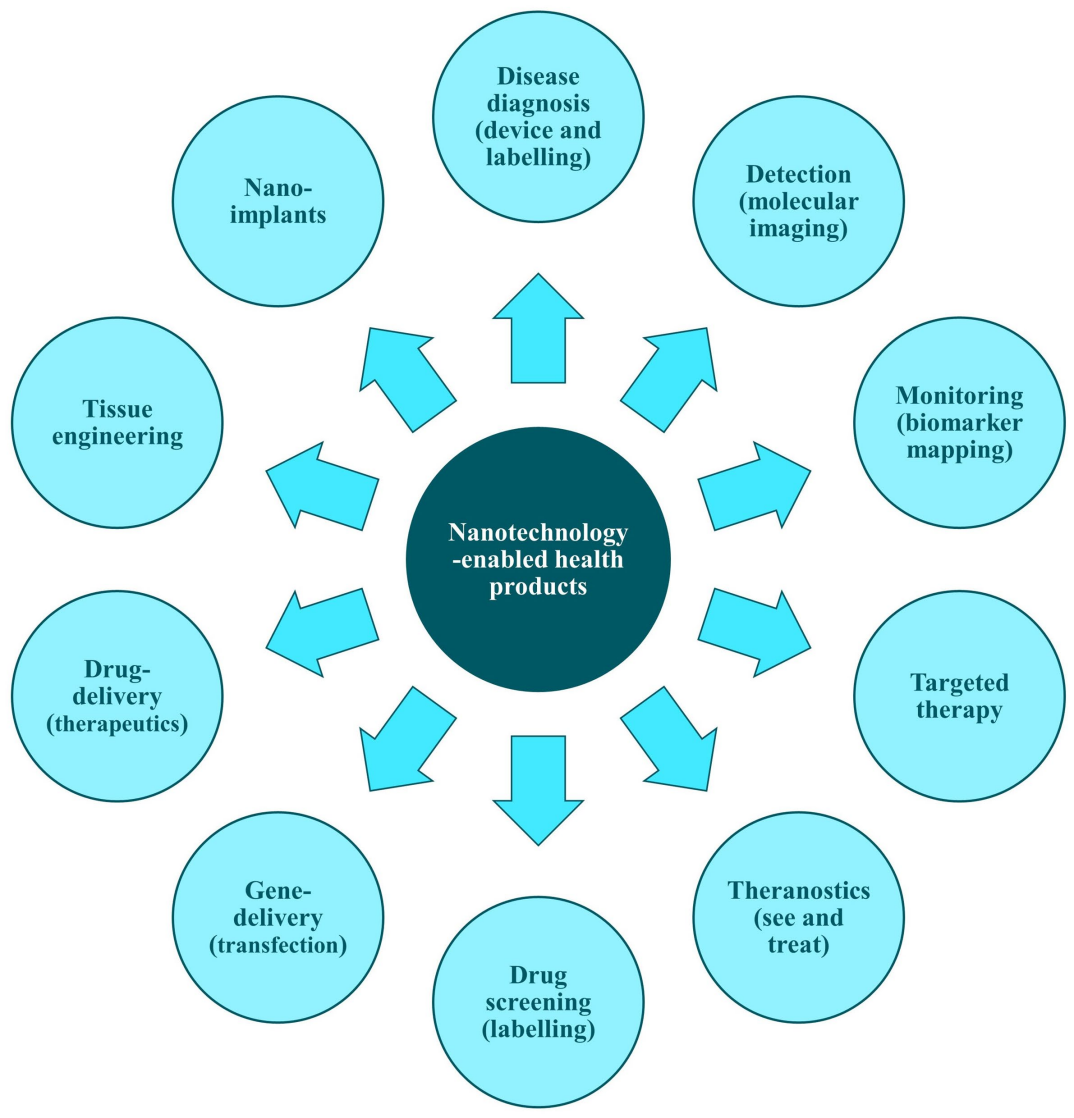
Examples of applications of nanotechnology-enabled health products. Adapted from ‘Figure 1’ of Acebes-Fernández et al. (101).

NHPs are designed to provide contrast in targeted areas and to convey information about the local environment upon introduction into the body. They facilitate tissue labelling with specific markers and enable the measurement of concentrations of targeted molecules, thus aiding direct disease analysis within the human body. Additionally, NHPs are used *in vitro* for the analysis of human proximal body fluids—key sources of biomarkers—supporting comprehensive diagnostic strategies for detecting molecular changes. They allow for the simultaneous analysis of multiple biomarkers, thereby enhancing diagnostic precision and reliability (Figure 3) (101).

Market forecasts indicate substantial growth in the global nanotechnology sector (28). Projections estimate an annual growth rate ranging from 9.2 to 36.4% up to 2030. The nanomaterials market, valued at 7.1 billion United States dollars (USD) in 2020, is anticipated to escalate to 13.60 billion USD by 2027. This surge is primarily attributed to the increasing demand for nanomaterials in health products, particularly drug delivery systems. The Asia-Pacific region is expected to experience the most rapid market expansion (29, 99).

## 2 NHP regulatory framework

### 2.1 Global regulatory overview

Health product regulations consist of complex legal, administrative, and technical measures implemented by governments to ensure the safety, efficacy, and quality of medicinal products and medical devices. They also verify that product information is relevant and accurate (30). These regulations vary according to the legislative frameworks specific to different geographic regions. Prominent global regulatory areas include:

- North America (NoA), which comprises Canada and the USA.
- Latin America (LATAM), encompassing all American countries outside of NoA.
- Europe, the Middle East, and Africa (EMEA).
- Asia-Pacific (APAC).

The regulatory systems of the EU and the USA are particularly influential, often setting the benchmark for international regulatory standards. In the EU, the European Commission (EC) provides the foundational legal framework, whereas in the USA, the FDA is the enforcing body for critical health product legislation. These jurisdictions serve as reference points for regulatory practices around the globe.

When it comes to regulatory classifications, NHPs are primarily categorized as either medicinal products or medical devices. The distinction between these categories is based on the product’s principal mechanism of action for achieving its intended purpose. Medicinal products are constructed to operate through pharmacological, immunological, or metabolic (PIM) mechanisms. Conversely, medical devices function predominantly through physical or mechanical means, although they may include PIM actions that supplement the primary physical mechanism.

Within the term ‘medical devices’, in addition to *in vivo* medical devices, there is also the regulatory category of *in vitro* diagnostic medical devices (IVDs). Due to their nature of use, these products do not present the same technical and safety challenges as those in contact with the human body (both medical devices and medicinal products). This work focuses primarily on highlighting the need to develop a clear regulatory framework for NHPs in contact with the human body. Nevertheless, it is also important to acknowledge the existence of significant regulatory challenges in the development of IVDs.

#### 2.1.1 Regulatory approval of medicinal products

##### 2.1.1.1 EU

The legislative framework for medicinal products in the EU is delineated by Directive 2001/83/EC. Article 1(2) within this directive offers a detailed definition of a medicinal product (31).

*Medicinal product:*

*(a) Any substance or combination of substances presented as having properties for treating or preventing disease in human beings; or.*

*(b) Any substance or combination of substances which may be used in or administered to human beings either with a view to restoring, correcting or modifying physiological functions by exerting a pharmacological, immunological or metabolic action, or to making a medical diagnosis.*

In the EU, obtaining marketing authorization is a prerequisite for all medicinal products, including those incorporating nanotechnologies, before they can be marketed and provided to patients. Nanomedicines may appear in various forms—some are informally referred to as non-biological complex drugs (NBCDs) due to their physicochemical complexity. Regardless of the exact categorization, this process requires the submission of a marketing authorization application (MAA), which is contingent upon the product’s regulatory classification. The MAA is reviewed under one of the following procedures: nationally within a single member state, through a decentralized procedure involving multiple states, or via a centralized procedure at the community level. Centralized review is obligatory for nanomedicines that are developed using certain biotechnological methods, aimed at treating serious diseases, or defined as orphan drugs (31). The national competent authorities (NCAs) conduct the MAA review for national and decentralized procedures, while the EMA is responsible for the centralized procedure.

Nanomedicines, as NBCDs, are intricate due to their physicochemical characteristics and lack a distinct legal classification within the EU. Consequently, they are regulated on a ‘case-by-case’ basis, without a dedicated regulatory pathway (32). This contrasts with biotechnology-derived medicinal products, which are required to follow the centralized procedure. The ambiguous regulatory landscape for NBCD follow-on products leaves uncertainty between adopting a ‘generic application’ under Article 10(1) or a ‘hybrid application’ under Article 10(3). Research indicates a trend towards the hybrid pathway (33).

**Figure 4:**
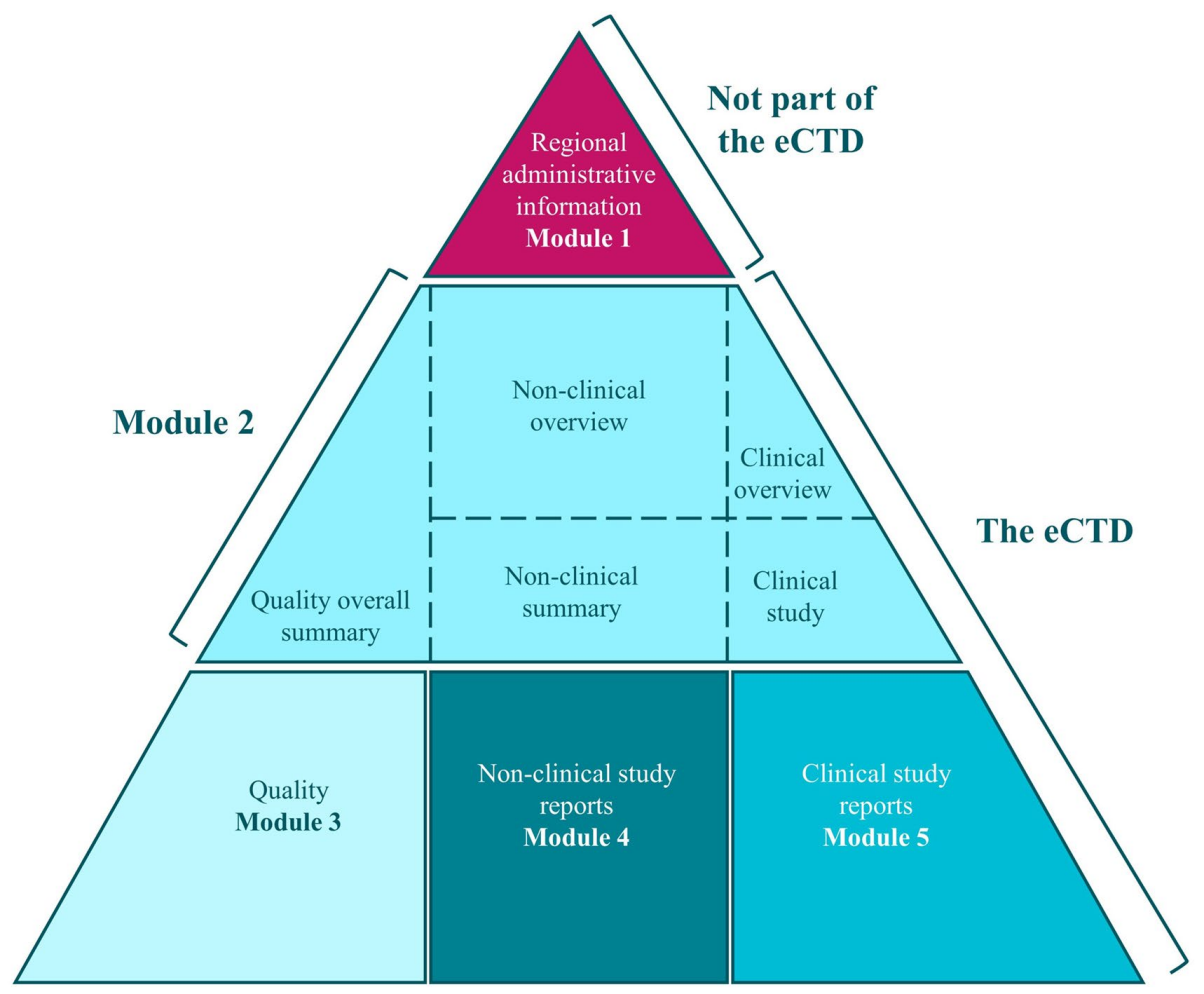
Electronic Common Technical Dossier (eCTD) format [taken from (34)].

The key legislative documents for submitting and reviewing MAAs in the EU are Directive 2001/83/EC and Regulation 726/2004/EC. These ensure that all medicinal products, including nanomedicines as NBCDs, are thoroughly evaluated for safety, efficacy, and quality. Applications must be submitted in the electronic common technical document (eCTD) format through the eSubmission gateway, in line with International Council for Harmonisation of Technical Requirements for Pharmaceuticals for Human Use (ICH) M4 guidelines, and applicants must be established within the European Community (Figure 4) (34).

The current EU pharmaceutical legislation has been instrumental in the authorization of safe and efficacious medicines. Nonetheless, it faces multiple challenges, including the need to ensure equitable access to these treatments and to maintain a steady supply. In recent years, the prevalence of medicinal shortages has increased, adversely affecting healthcare systems and compromising patient care.

Although the existing legislative framework fosters innovation and supports the development of novel treatments, there is a notable disparity in patient access across Member States. Furthermore, the pace of innovation often fails to match unmet medical needs—particularly in the context of antimicrobial development (35). In response, the EC has proposed reforms that aim to bolster health protection standards, guarantee EU-wide access to medicines, and safeguard supplies. These reforms seek to update the legislation in line with current scientific advancements and to reduce the environmental impact of pharmaceuticals, forming part of a wider strategy to enhance public health, market supervision, and the accessibility of essential medicines. Fostering innovation and competitiveness in the EU market, while ensuring environmental sustainability, is deemed crucial, particularly for rare diseases and pediatric medicines (35).

Despite the breadth of these reforms, the new European Pharmaceutical Legislation (35) does not yet provide explicit provisions for nanotechnology-enabled health products. Introducing clearer regulatory pathways or guidance for such products could offer greater transparency, especially for complex and hybrid applications, and could help streamline the authorization process across Member States. The Centralized Procedure, in particular, may confer notable advantages by reducing administrative duplication and ensuring greater consistency in their evaluation and approval. Addressing these considerations in the legislative debates holds the potential to accelerate development and expand patient access to innovative nanotechnology therapies more efficiently.

##### 2.1.1.2 United States

In the USA, the definition of medicinal product is set forth in section 201(g) of the Federal Food, Drug, and Cosmetic Act (FD&C Act) (36):

*The term ‘drug’ means:*

*(a) Articles recognized in the official United States Pharmacopoeia, official Homoeopathic Pharmacopoeia of the United States, or official National Formulary, or any supplement to any of them; and.*

*(b) Articles intended for use in the diagnosis, cure, mitigation, treatment, or prevention, of a disease in man o other animals; and.*

*(c) Articles (other than food) intended to affect the structure or any function of the body of man or other animals; and.*

*(d) Articles intended for use as a component of any article specified in clause (a), (b), or (c).*

*A food or dietary supplement for which a claim, subject to sections 343(r)(1)(B) and 343(r)(3) of this title or sections 343(r)(1)(B) and 343(r)(5)(D) of this title, is made in accordance with the requirements of section 343(r) of this title is not a drug solely because the label or the labeling contains such a claim.*

*A food, dietary ingredient, or dietary supplement for which a truthful and not misleading statement is made in accordance with section 343(r)(6) of this title is not a drug under clause (C) solely because the label or the labeling contains such a statement.*

The FDA is the competent authority tasked with the evaluation and approval of pharmaceuticals in the United States. It is a vast governmental institution composed of various offices, each specialising in a different regulatory category of products (FDA organization chart can be consulted in the following website: https://www.fda.gov/media/171675/download?attachment). Within the FDA, the Center for Drug Evaluation and Research (CDER) and the Center for Biologics Evaluation and Research (CBER) play crucial roles in drug regulation.

- The CDER’s Office of New Drugs (OND) assesses new pharmaceuticals prior to their market release, while the Office of Drug Safety (ODS) monitors post-market drug safety.
- The CBER’s Office of Tissues and Advanced Therapies (OTAT) oversees advanced therapies such as cell and gene treatments. These offices work collaboratively to ensure that medications are both safe and effective, which is a fundamental principle in the science of drug regulation.

The process for applying to the FDA for marketing authorisation (MA) of a new drug based on a chemical active substance is known as a ‘New Drug Application’ (NDA). The FDA delineates three types of NDAs in Part 314 of Title 21 of the Code of Federal Regulations (21 CFR 314), which interprets the FD&C Act and related statutes, forming the legal foundation for food, medical device, and drug legislation in the USA. This law’s primary goal is to guarantee that medical products and drugs are, in that order, safe and effective for their intended end-users.

A brief outline of the regulatory pathways for an NDA submission includes:

- Section 505(b)(1): an application comprising complete reports of investigations on safety and effectiveness, used for drugs with active ingredients never before approved.
- Section 505(b)(2): an application including complete reports on safety and effectiveness, but where some approval information comes from studies not conducted by or for the applicant without a right of reference.

In addition to the NDA process, there exists a separate pathway for biological products known as ‘Biologics License Applications’ (BLA). Through the CBER, the FDA reviews BLAs to ensure that the biological products meet the necessary criteria for purity, safety, and efficacy. The BLA is a comprehensive document that must include data from preclinical and clinical studies, demonstrating that the biologic is safe and effective for its intended use. The CBER’s rigorous evaluation process ensures that these complex products meet the standards required to protect public health.

For submitting an application for a product that is a duplicate of a previously approved medicinal product, the legal basis is referred to as ‘abbreviated new drug application’ (ANDA) and it is described in section 505(j). It consists of an application providing evidence that the proposed product is identical to a previously approved product in active ingredient, dosage form, strength, route of administration, labelling, quality, performance characteristics, and intended use, among other attributes.

It is important to emphasise that, as mentioned, under the 505(b)(1) pathway, all studies to demonstrate the final product’s quality, safety, and efficacy must be conducted by the applicant or must have the right to reference third-party results.

Similar to the EU, in the USA, nanomedicines may be referred to as NBCDs and do not have specific legal bases, leading to a ‘case-by-case’ approach for their conformity assessment (32). New nanomedicine products may follow any of the NDA routes. For follow-on products, the FDA recommends the 505(j) ANDA pathway as the standard evaluation route for complex generic drug products, including those containing nanomaterials. This pathway allows for the approval of a generic drug based on bioequivalence to the reference product (37).

The general list of studies and requirements are described in the legislative text of this legal basis and are essentially the same as those for a complete dossier submitted in the EU according to Article 8(3) of Directive 2001/83/EC.

#### 2.1.2 Regulatory approval of medical devices

##### 2.1.2.1 EU

In the EU, medical devices are governed by Regulation (EU) (38) (commonly referred to as ‘Medical Device Regulation’ or MDR). Article 2(1) within this regulation includes a detailed definition of medical device:

“*Medical device’ means any instrument, apparatus, appliance, software, implant, reagent, material or other article intended by the manufacturer to be used, alone or in combination, for human beings for one or more of the following specific medical purposes:*

*diagnosis, prevention, monitoring, prediction, prognosis, treatment or alleviation of disease,*

- *diagnosis, monitoring, treatment, alleviation of, or compensation for, an injury or disability,*
- *investigation, replacement or modification of the anatomy or of a physiological or pathological process or state,*
- *providing information by means of in vitro examination of specimens derived from the human body, including organ, blood and tissue donations,*

*and which does not achieve its principal intended action by pharmacological, immunological or metabolic means, in or on the human body, but which may be assisted in its function by such means*’.

For enhanced clarity on the definition of a medical device, specifically regarding its primary mode of action, the Medical Device Coordination Group (MDCG), established in accordance with Article 103 of the MDR, has released guidance MDCG 2022-5. This guidance offers further explanation on actions that operate on pharmacological, immunological, or metabolic (PIM) means (Table 1) (29).

**Table 1:** Definition of pharmacological, immunological, and metabolic means as defined in MDCG 2022-5.

Action	Definition
Pharmacological	*‘Pharmacological means’ is understood as an interaction typically at a molecular level between a substance or its metabolites and a constituent of the human body which results in initiation, enhancement, reduction or blockade of physiological functions or pathological processes.*
Immunological	*‘Immunological means’ is understood as an action initiated by a substance or its metabolites on the human body and mediated or exerted (*i.e.*, stimulation, modulation, blocking, replacement) by cells or molecules involved in the functioning of the immune system (*e.g.*, lymphocytes, toll-like receptors, complement factors, cytokines, antibodies).*
Metabolic	*‘Metabolic means’ is understood as an action of a substance or its metabolites which involves an alteration, including stopping, starting or changing the rate, extent or nature of a biochemical process, whether physiological or pathological, participating in, and available for, function of the human body.*

In the EU, for a medical device to be legally sold, it must obtain a CE mark (*Conformité Européene*). This mark indicates that the device meets the safety and performance requirements according to the MDR, depending on its intended use as stated by the manufacturer. The general safety and performance requirements (GSPRs) are listed in Annex I of the MDR. Identifying which of these requirements apply to a specific medical device early in the development process is fundamental. This ensures that the necessary steps are taken to demonstrate the device’s compliance through various tests and studies. The data collected to show compliance is compiled in a file called the Technical Documentation (39).

Additionally, a medical device’s compliance is linked to the manufacturer’s Quality Management System (QMS). The QMS’s processes ensure that the device consistently meets standards at all stages of production and throughout its lifespan (39).

For the CE marking of certain medical devices in the EU, they must be evaluated by a conformity assessment body designated in accordance with the MDR, as applicable, that is, a Notified Body (NB). A NB is an entity appointed by an EU member state to assess the conformity of certain risk classes of devices. The NB evaluates both the Technical Documentation and the QMS to ascertain conformity with the MDR, following the selected conformity assessment route (39).

Medical devices regulated under the MDR are categorized into four risk classes (I, IIa, IIb, and III), based primarily on the intended use of the product. The classification also considers factors such as the invasiveness of the product, the duration and nature of patient contact, and other special characteristics. There are 22 classification rules in total, defined in Annex VIII of the MDR. Notably, the MDR introduces a specific classification rule for products that incorporate nanomaterials within their composition, Rule 19 (39):

*All devices incorporating or consisting of nanomaterial are classified as:*

- *class III if they present a high or medium potential for internal exposure;*
- *class IIb if they present a low potential for internal exposure; and*
- *class IIa if they present a negligible potential for internal exposure.*

**Figure 5:**
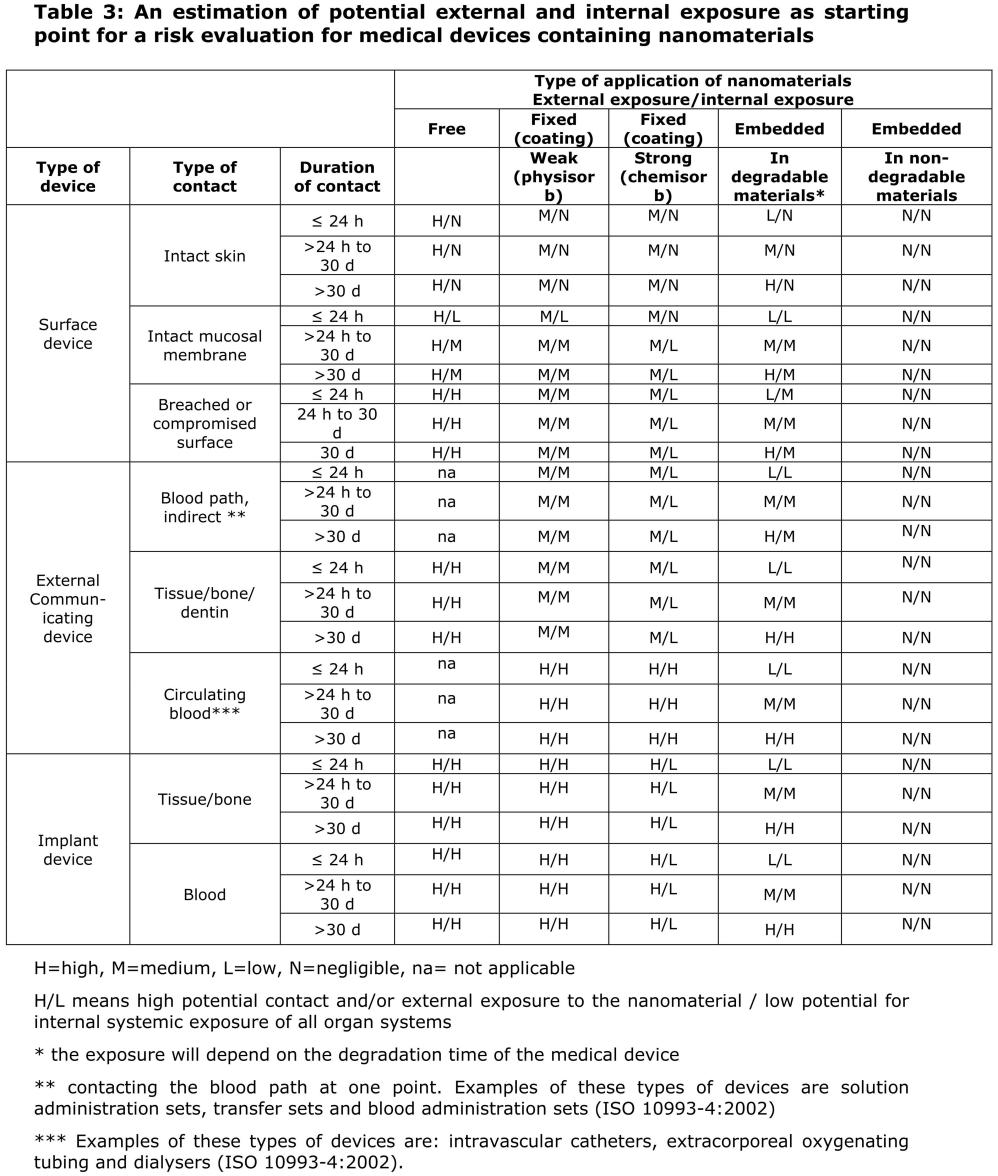
Extract from SCENIHR guidance for the determination of potential of internal exposure to nanomaterials in medical devices.

In the EU, NHPs regulated as medical devices are always classified, at a minimum, as Class IIa; therefore, the conformity assessment always requires the involvement of an NB. To facilitate the correct application of this rule, the Scientific Committee on Emerging and Newly Identified Health Risks (SCENIHR) published specific guidance for determining the potential for internal exposure based on the type of nanomaterial application (free, fixed in a coating, or embedded), the type of contact with the body, and the nature of this contact (Figure 5) (40).

As previously mentioned, the significance of risk class classification is inherently linked to the applicable requirements for the corresponding conformity assessment process. Products within higher risk classes require a more comprehensive conformity assessment process, conducted by NBs. Conversely, products within the lowest risk class, Class I, can have their conformity assessment conducted by the manufacturer itself and can be placed on the market without the involvement of an NB (39).

##### 2.1.2.2 United States

In the USA, the FDA is the regulatory authority responsible for the regulation of medical devices. It falls under the jurisdiction of the Center for Devices and Radiological Health (CDRH). The statutory definition of a medical device is provided in Section 201(h) of the Federal Food, Drug, and Cosmetic Act (FD&C Act) (36):

*The term ‘device’ means an instrument, apparatus, implement, machine, contrivance, implant, in vivo reagent, or other similar or related article, including any component, part, or accessory, which is:*

- *Recognized in the official National Formulary, or the United States Pharmacopeia, or any supplement to them,*
- *Intended for use in the diagnosis of disease or other conditions, or in the cure, mitigation, treatment, or prevention of disease, in man or other animals, or*
- *Intended to affect the structure or any function of the body of man or other animals,*

*And which does not achieve its primary indications for use through chemical action within or on the body of man or other animals and which is not dependent upon being metabolized for the achievement of its primary indications for uses.*

The FDA categorizes medical devices into three risk-based classes: Class I, Class II, and Class III, which range from low to high risk, respectively. Similar to the EU, the classification system is intrinsically linked to the level of regulatory oversight required to ensure the safety and efficacy of these devices.

In the USA, regulatory requirements of safety and efficacy are referred to as ‘General and Special Controls’. Identifying the relevant controls for a new device at the earliest stages of development is critical to ensure compliance with regulatory standards and to expedite the product’s time to market.

The pathway to commercialization of medical devices in the USA may require an FDA review. While most Class I devices and certain Class II devices are exempt, the majority of Class II devices and all Class III devices must undergo FDA scrutiny. There are three primary FDA review processes for the registration and commercialization of medical devices in the USA:

- The Premarket Notification [510(k)] process requires a submission to the FDA to demonstrate that the device in question is ‘Substantially Equivalent’ (SE) to a legally marketed device, known as a ‘predicate’. This involves proving not only SE to the predicate device but also compliance with the applicable general and special controls.
- The Premarket Approval (PMA) is a more rigorous process involving scientific and regulatory review to assess the safety and effectiveness of Class III devices, which goes beyond general and special controls.
- The *De Novo* Submission, or ‘*De Novo* process’ [513(f)], provides a pathway for classifying novel, moderate risk devices that lack a legally marketed predicate device. In cases where general controls, or a combination of general and special controls, are sufficient to ensure safety and effectiveness for their intended use, the *De Novo* process can be used for risk-based down-classification of the device.

The specifics of the Product Dossier are dictated by the chosen FDA review procedure:

- A 510(k) dossier is compiled to establish SE with a predicate device.
- A PMA dossier represents the most comprehensive Product Dossier, including complete Clinical Evidence data.
- A *De Novo* dossier aims to justify the absence of a suitable predicate and to demonstrate the moderate risk associated with the device’s use.

As with the majority of medical devices, for NHPs regulated as medical devices, the PMA pathway is the least common. According to the study by Jones AD, a search of the FDA’s database of medical devices sold in the USA [ACCESS Global Unique Device Identification (GUDID)] from 1980 to 2017 revealed 2.586 ‘nano* implantable devices’ from a total of 16 unique manufacturers (the symbol ‘*’ is used for truncation of search terms). Of those, only 36 had to go through the lengthier PMA process. Multiple filings by the same manufacturer may indicate either that manufacturers are taking advantage of ‘serial predicates’, which raises important questions regarding the effectiveness of regulatory oversight in fostering innovation while protecting public health, or that there are prohibitive challenges (41).

Finally, as in the EU, medical device manufacturers in the USA are obliged to implement a QMS in accordance with the Quality System Regulations (QSR)(21 CFR 820) (36).

### 2.2 Regulatory consultations procedures

#### 2.2.1 Medicinal products

##### 2.2.1.1 EU

###### 2.2.1.1.1 Scientific advice procedure with EMA

Scientific Advice (SA) is the process by which a competent authority gives advice to an applicant during the development of a product, and is prospective in nature. SA questions can relate to the suitability of quality, nonclinical and clinical development programmes including specific issues such as drug substance / product specifications, design and rationale for non-clinical toxicity studies and clinical trial design features. In general, SA meetings are used to request Agency endorsement or feedback for proposed exceptions, justifications, marginal issues or aspects of development not clearly covered by existing regulatory guidance.

The SA procedure at EMA is generally ‘high level’, in the sense that there is a greater focus on the final MAA and a certain level of expectation regarding (i) quality and breadth of available data and (ii) the potential of the product. In addition, the EMA does not approve clinical trials which is done at the national level, so questions regarding suitability of documentation for clinical trial authorisation are best directed at the appropriate NCAs although EMA may comment on the suitability of overall clinical trial design to support MAA.

Companies can request SA from EMA at any stage in product development. At the technical level, EMA SA in the form of a written document is issued by the Committee for Medicinal Products for Human Use (CHMP) on the recommendation of the Scientific Advice Working Party (SAWP).

Some additional points to consider are:

- SA is not mandatory, but it is highly recommended and can be conducted on as many occasions as necessary; it is worth noting that there is a general sense that drug developers that work in collaboration with regulators maximise the probability of a successful MAA (42).
- Optimal times for SA procedures are:
  - Before initiation of first-in human studies to ensure that the Quality and Nonclinical development programs are adequate to support proposed early phase clinical trials and pivotal (in compliance with good laboratory practices) toxicity studies, and,
  - Prior to late-stage clinical trials to ensure that the study design(s) are adequate and that the completed Quality and Nonclinical packages to be submitted are sufficient to support marketing and to address regulatory issues related to the MAA.

###### 2.2.1.1.2 ITF meeting

The EMA’s Innovation Task Force (ITF) is a multi-disciplinary group that includes experts with scientific, regulatory and legal competencies. Meetings with ITF provide a forum for early dialogue with Applicants on innovative aspects in product development. An ITF meeting offers an early engagement platform for research and development aimed towards innovative therapeutic approaches. The meetings allow interaction at any early stage in development and a broad-ranging general discussion where opinions can be exchanged in a relatively informal setting. At such meetings, the Company makes a presentation of their development plan and general feedback on the development program is given (e.g., regarding the acceptability of proposed animal models).

ITF briefing meetings are free of charge and are usually held within approximately 60 days of receipt of a valid application. The first step in the process is to fill out a short request form, detailing the product and the topics to be discussed (which can relate to quality, nonclinical or clinical development) to be followed by a more detailed briefing document containing a series of suggested topics for discussion at the meeting along with background information about the product. Detailed questions on specific aspects of development are normally considered to be out of the scope of the process, which is described by the ITF Secretariat as ‘*joint brainstorming on innovative methods, technologies and products linked to drug development.*’

The ITF is open for discussion on any issue related to the development of emerging therapies and technologies, including nanomedicines, even if the regulatory classification of the intended product is not immediately evident. The outcomes of the meetings with the ITF are specific for early-stage development of complex projects and are non-binding. An advantage compared to SA is that the applicant does not need to have a position statement on the questions raised, and no fees apply to this procedure. Therefore, ITF meetings are an excellent opportunity to obtain informal and preliminary scientific advice, particularly when exploring theoretical discussions with less concrete information compared to what is required for formal scientific advice procedures. For more detailed answers to specific questions, a SA or Qualification Advice meeting would be more appropriate. Notably, nanotechnologies, including nanomedicines, have been among the top 10 themes discussed in ITF meetings in recent years, present in 5% of the procedures from 2019 to 2022 (43).

ITF meetings are generally considered a useful way of preparing for, offering insights from EMA experts on proposed approaches. It should be noted that these meetings are granted by the ITF Secretariat at their discretion based on the submission of a meeting request and a short draft briefing document – the ITF Secretariat may also select only some of the proposed topics for discussion at the meeting, depending on whether they are considered in or out of scope.

###### 2.2.1.1.3 SME briefing meeting

Small and medium-sized companies (SME) Briefing Meetings are offered by the EMA’s SME office. The function of these meetings is to give guidance to companies on their regulatory strategy (but not necessarily scientific or technical questions). To do these meetings, the SME office require some background information of the product and the stage of development as well as the questions the Company would like to raise.

These meetings are not considered replacements for the ITF or SA meetings and there is no limit in the number of meetings. The meetings normally last 90 min and the format of these meetings consists of a 15–20-min presentation by the company to present the background of the product development, with the rest of the time available for questions. The SME briefing meetings count with the participation of EMA staff from different offices (e.g., scientific advice, orphans, pediatrics, regulatory affairs). It may be useful to conduct this type of meeting first to ensure the maximum benefit is obtained from the ITF meeting.

###### 2.2.1.1.4 Scientific advice with NCAs

Several European NCAs offer informal opportunities for early-stage dialogue. For example, the Paul-Ehrlich-Institut (PEI) Innovation Office in Germany also offers Pre-Advice meetings for products in a very early stage of development for general (scientific) guidance and regulatory orientation and is an informal exchange on general issues. These meetings are also in addition to standard scientific advice meetings. As PEI is an international leader on biological products and their manufacture, this NCA can be an interesting choice for discussion.

Also, of note, the EMA and European Heads of Medicines Agencies (HMA) have initiated the second phase of Simultaneous National Scientific Advice (SNSA) pilot program that is due to run until late 2024. This permits SA to be conducted with up to 3 NCAs (maximum of two participating NCAs and one observer NCA) simultaneously via a single common application procedure, and with a consolidated final advice incorporating the positions of each NCA.

After the withdrawal of the UK from the EU, there may also be scope for consultations with the Medicines and Healthcare Products Regulatory Agency (MHRA) at different stages in product development via the MHRA Innovation Office and Scientific Advice service. There is also the possibility of joint meetings with the MHRA and the National Institute for Healthcare and Excellence (NICE), which may be useful for the discussion of later stage (e.g., Phase III) clinical study design.

##### 2.2.1.2 United States

The FDA offers three types of formal meetings for developers of medicinal products, which are classified as Type A, Type B and Type C meetings, as described below and in the relevant FDA guidance (44):

- Type A Meeting: these meetings aim to provide assistance to a stalled product development program (e.g., to discuss a new path forward after responses to a clinical hold) or to provide ‘Special protocol assessment’, which is a process designed to achieve agreement between developers and FDA with regard to critical design elements of clinical and / or nonclinical studies considered critical for supporting marketing approval (45).
- Type B Meeting: these meetings are scheduled at specific points (‘milestones’) during the development program; just before submission of a first-in human study, at the end of phase 1 of clinical development (End-of-Phase 1 meeting), at the end of phase II / start of phase III of clinical development (End-of-Phase 2 meeting and pre-Phase III meeting), and just prior to submission of the BLA (pre-BLA meeting). In general, Type B meetings are broadly similar in format and purpose to the SA procedures with EMA.
- Type C Meeting: any other meeting with the FDA regarding the development and review of a product that is not a Type A or Type B meeting.

Type D Meeting: these meetings are focused on a narrow set of issues (should be limited to no more than 2 focused topics) and should not require input from more than 3 disciplines or Divisions.

#### 2.2.2 Medical devices

##### 2.2.2.1 EU

In the EU, as previously mentioned, the authorities responsible for issuing CE marking for products above Class I are the NBs. These institutions are not authorized to provide consultancy services during the development of medical devices to manufacturers. Therefore, medical device manufacturers must approach NBs once they have nearly completed the development of their product and are ready to submit the technical documentation.

For innovative medical devices such as some NHPs, the pilot program for Scientific Advice for High-risk Medical Devices may be of interest. This program, in line with Article 61(2) of the MDR, is open to all manufacturers established in the EU and aims to provide support related to clinical development strategy and the proposal of clinical investigations for Class III and active Class IIb MD intended for the administration of medicines. Applications from small and medium-sized enterprises receive special attention to ensure their representation in the program. The third phase of the program finished on April 30th, 2024; new editions of the program are anticipated in the future.

##### 2.2.2.2 United States

In the USA, however, there is the Q-Submission Program, a more established program that offers various mechanisms for requesting feedback from the FDA at different stages of development and after submitting the product’s PMA.

- Pre-submission: a formal request for comments through which the FDA helps guide product development and/or the preparation of the application.
- Informational meetings: the sponsor shares information with the FDA without expecting comments; the FDA will be in ‘listening’ mode. This is appropriate if the sponsor wants to familiarize the FDA review team with a new device that has significant technological differences from current devices. It is also useful for providing an overview of development when multiple submissions are planned.
- Study risk determination: to request the FDA to determine if a clinical study is of significant risk or non-significant risk.
- Submission issue request: a request for FDA feedback to address issues communicated in a hold letter from a marketing application.

It is highly recommended, especially for such novel products with few precedents and applicable regulatory guidelines, to take advantage of these consultation procedures. They can ensure optimal product development in accordance with FDA expectations, the authority responsible for the final product authorization.

## 3 Regulatory state of the art

To demonstrate compliance with the applicable regulatory requirements for NHPs, it is critical to consider the state of the art in both technology and regulatory affairs.

The term ‘state of the art’ is frequently used to denote the most modern or advanced stage of development in a specific field, reflecting the highest level of development from the latest techniques and technologies. It is also defined as the level of knowledge and development achieved in a particular technique or science, particularly in contemporary times (46). In the context of patent law, the state of the art is the collection of all technical knowledge that has been made publicly available (47).

**Figure 6:**
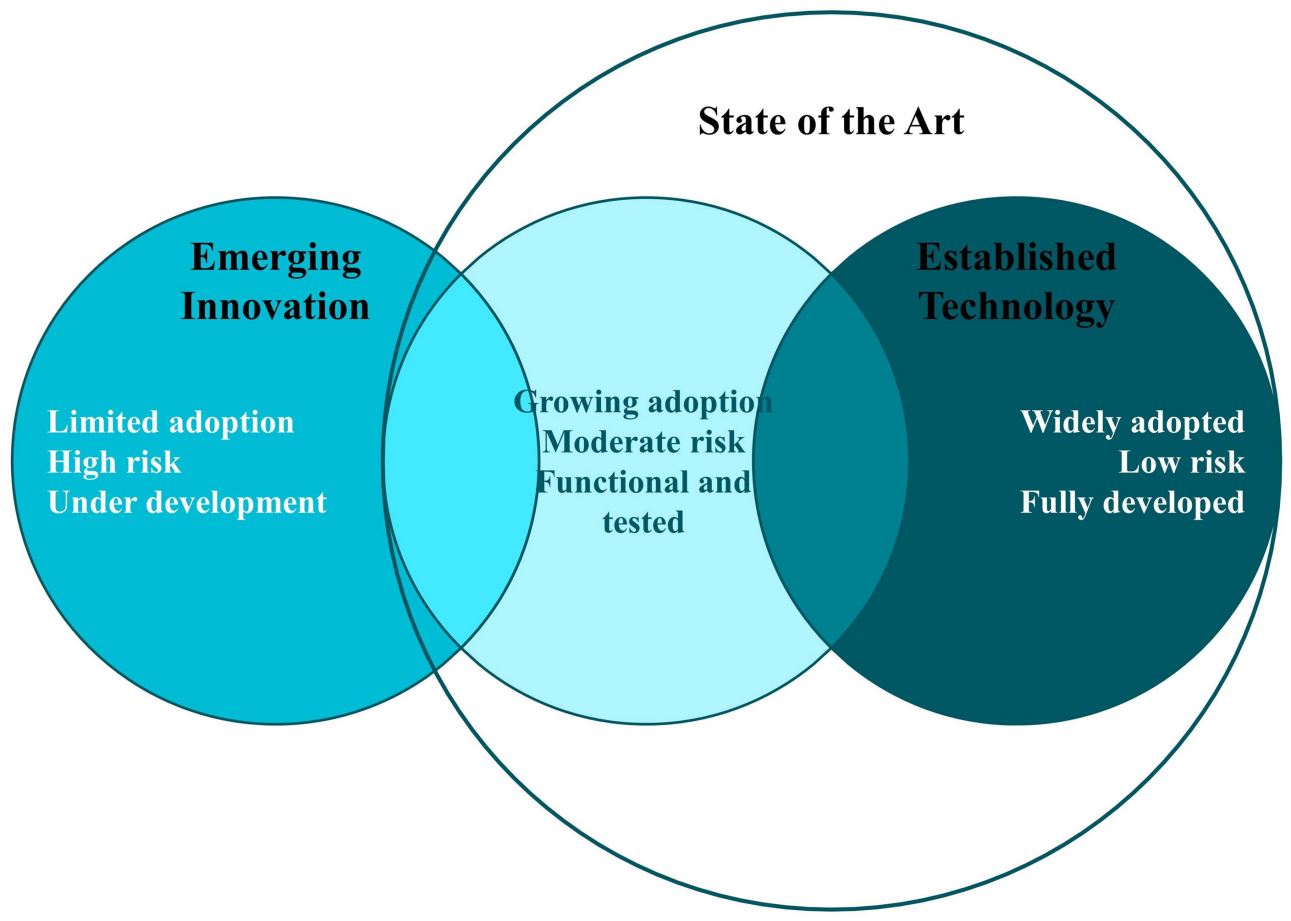
State of the art from a regulatory perspective.

From a regulatory standpoint, the state of the art in technology indicates the current level of technical development in terms of products, processes, and services, which is grounded in the established findings of science, technology, and accumulated experience. This concept is what is currently accepted as good practice in the fields of technology and medicine, and it is essential for establishing the baseline from which to demonstrate the quality, safety, and efficacy of health technologies, including NHPs. However, it is important to note that the state of the art does not necessarily correspond to the most advanced technological solution (48) (Figure 6).

The regulatory state of the art, meanwhile, encompasses the most recent technical standards, relevant legislation, and current regulatory guidelines. These elements together represent the most advanced and recognized level in the field of product-specific regulation (49). Such an integrated perspective ensures that the regulatory state of the art is in sync with the technological state of the art, encouraging the creation of products that are innovative and adhere to the highest standards of safety and efficacy.

### 3.1 Regulatory state of the art for NHPs regulated as medicinal products

For NHPs regulated as medicinal products, the legislative framework sets out specific requirements that must be fulfilled. MA holders must comply with a variety of general and specific pharmacopeia chapters and guidelines to ensure the product’s quality, safety, and efficacy.

The globalization of medicinal product development has prompted regulators to focus on shared approaches and standards, while preserving autonomy in decision-making within their legal mandates. The ICH is instrumental in harmonizing international regulatory standards for medicines, thereby facilitating a coherent global regulatory framework in the pharmaceutical industry. The ICH guidelines are divided into themes of quality, safety, efficacy, and multidisciplinary guidelines.

There are also voluntary international forums with varying objectives: the International Conference of Drug Regulatory Authorities (ICDRA), backed by the World Health Organisation (WHO), aims to discuss priorities and strengthen convergence, especially for low- and middle-income countries; and the International Coalition of Medicines Regulatory Authorities (ICMRA) was formed by agency heads for executive-level exchanges (50).

Although ICH guidelines are generally applicable to pharmaceuticals, they do not always include specific considerations for NHPs, particularly those using nanotechnology, and may not be directly applicable. For instance, genotoxicity tests recommended by regulatory bodies may yield false-negative results for nanomaterials, leading to an underestimation of their mutagenic effects on cells. In relation to genotoxicity, the *in vitro* Ames test assay, which has produced false negatives when tested with nanomaterials (as nanomaterials sometimes do not penetrate the bacterium and thus have no effect), is deemed unsuitable for evaluating mutagenicity. Carcinogenicity studies, required only when the drug is administered over a long period, are lengthy, typically taking around 2 years. For certain nanomaterials, such as nickel-based ones, the mechanisms underlying carcinogenicity are not entirely understood, necessitating the development and validation of specific methods. Concerning immunotoxicity, it is clear that nanomaterials can interact with the immune system, potentially causing immune responses that affect the medicinal product’s efficacy and/or safety. Notably, instances of CARPA syndrome (a pseudo-allergy syndrome associated with complement C-activation) have been documented with various nanomaterial systems. Currently, there is an absence of a specific regulatory framework, and there is a need for the development of new models that may eventually establish new regulatory standards. This underscores the need for specific guidelines tailored to the unique properties of these products (51).

### 3.2 Regulatory state of the art for NHPs regulated as medical devices

The demonstration of conformity with regulatory requirements for medical devices, including NHPs, in the EU and the USA relies on adhering to guidelines issued by competent authorities, international standards, and literature references, as well as the application of proprietary methodologies and their validation, or a combination of these various approaches.

Standard-emitting organizations such as ISO and ASTM, through their specialized working groups, are pivotal in developing and updating standards that pertain to the medical device industry. There are ISO and ASTM standards that address a wide range of aspects in medical device development, from quality considerations and risk management to preclinical and clinical evaluations. Within these organizations, there are committees specifically dedicated to NHPs, such as ISO/TC 229 Nanotechnologies and ASTM’s E56.08 on Nano-enabled medical products.

In the EU, the European committees CEN (European Committee for Standardization) and CENELEC (European Committee for Electrotechnical Standardization) review ISO standards to ensure they align with the requirements of the MDR. Upon endorsement, these standards are published in the Official Journal of the EU as ‘harmonized’ standards and include an annex, Annex Z, which outlines their correlation with the legal mandates of the MDR. While adherence to these standards is voluntary, products that comply with the relevant harmonized standards are presumed to satisfy the applicable GSPRs of the MDR (‘*presumption of conformity*’).

In the USA, the FDA reviews and adopts standards from various organizations, including but not limited to ISO and ASTM, and designates them as consensus standards. Declarations of conformity by the manufacturers to these standards are recognized as evidence of compliance with the relevant General and Special Controls.

Although not harmonized or designated as consensus standards, standards specifically relevant to NHPs are ISO/TR 13014:2012(E) *Guidance on physico-chemical characterization of engineered nanoscale materials for toxicological assessment* and ISO/TR 10993–22:2017 *Biological evaluation of medical devices—Part 22: Guidance on nanomaterials*. These standards provide guidance on physicochemical characterization methods for toxicological assessment. They aim to aid in assessing the toxicological impact of manufactured nano-objects, allowing for differentiation between materials that may appear similar. ISO/TR 10993–22:2017 also includes details on sample preparation and release toxicokinetic studies, covering absorption, distribution, metabolism, and excretion/elimination (38, 52). Despite this, current evaluations and models tend to overlook the consideration of ageing in medical devices, even though this aspect is included in the ISO 10993-22 standard. The current models for analyzing nanoparticle release from medical devices do not adequately address the ageing factor of these devices, despite comprehensive detailing of nanoparticle characterization in the ISO 10993-22 standard. Furthermore, existing models do not sufficiently consider the specific implantation site of medical devices within the body, the surrounding environment, or the duration of its implantation. Therefore, a tailored, case-by-case approach remains necessary for assessing the risks associated with medical devices, as there are no validated test methods specifically designed for nanomaterials at present. Consequently, these tests will need to be applied individually, with appropriate modifications considering the unique characteristics of nanomaterials based on their intended applications.

Furthermore, specific regulatory guidelines have been released by entities such as the MDCG in the EU and the FDA in the USA. There are also guidelines with global reference published by the International Medical Device Regulators Forum (IMDRF), the WHO, and the Organisation for Economic Co-operation and Development (OECD). The field of NHPs has seen an increase in the publication of specific guidelines for these medical devices.

## 4 Discussion: regulatory hurdles for NHPs

The regulation of health technologies consistently lags behind rapid advancements in research and development. This is particularly true for NHPs, where the delay in establishing specific regulatory guidelines is pronounced due to the unique properties of nanomaterials compared to their larger-scale counterparts. This delay has resulted in the ‘valley of death’, a term used to describe a significant decline in the number of NHPs that reach advanced stages of development, such as clinical trials or market release, relative to those in the research and development phase (53–55).

Several regulatory challenges exacerbate this ‘valley of death’, especially as progress in NHPs continues. These challenges include the lack of harmonized definitions, the paradigm of physicochemical characterization, and, specifically for products that come into contact with the human body, the difficulty in obtaining evidence related to biological safety, nanotoxicity, and *in vivo* behavior of NHPs (56–58).

### 4.1 Lack of harmonised definitions

Comparative analysis between similar products is vital in regulatory science, helping regulators and competent authorities to identify potential requirements for novel products based on existing ones. This global approach is useful even when comparing products approved under different regulatory jurisdictions, such as NHPs in the EU and the USA. However, a cornerstone for regulatory interoperability is the establishment of clear and harmonized definitions that allow for the consistent categorization of NHPs.

Within the EU, various legislative frameworks have their own definitions of nanomaterials (Table 2). Additionally, the EC published on 10 June 2022 the Commission Recommendation 2022/C 229/01, which includes an updated definition of nanomaterial (59):

**Table 2:** Nanomaterial definitions included in EU legal frameworks.

**Legislative framework**	**Definition**
Medical Device Regulation (EU) 2017/745	‘Nanomaterial’ means a natural, incidental or manufactured material containing particles in an unbound state or as an aggregate or as an agglomerate and where, for 50% or more of the particles in the number size distribution, one or more external dimensions is in the size range 1–100 nm.
Cosmetic Product Regulation (EU) 1223/2009	‘Nanomaterial’ means an insoluble or biopersistant and intentionally manufactured material with one or more external dimensions, or an internal structure, on the scale from 1 to 100 nm.
Novel food Regulation (EU) 1169/2011	‘Engineered nanomaterial’ means any intentionally produced material that has one or more dimensions of the order of 100 nm or less or that is composed of discrete functional parts, either internally or at the surface, many of which have one or more dimensions of the order of 100 nm or less, including structures, agglomerates or aggregates, which may have a size above the order of 100 nm but retain properties that are characteristic of the nanoscale. Properties that are characteristic of the nanoscale include: (i) those related to the large specific surface area of the materials considered; and/or (ii) specific physico-chemical properties that are different from those of the non-nanoform of the same material.
Biocidal product Regulation (EU) 528/2012	‘Nanomaterial’ means a natural or manufactured active substance or non-active substance containing particles, in an unbound state or as an aggregate or as an agglomerate and where, for 50% or more of the particles in the number size distribution, one or more external dimensions is in the size range 1–100 nm. Fullerenes, graphene flakes and single-wall carbon nanotubes with one or more external dimensions below 1 nm shall be considered as nanomaterials. For the purposes of the definition of nanomaterial, ‘particle’, ‘agglomerate’ and ‘aggregate’ are defined as follows: ‘particle’ means a minute piece of matter with defined physical boundaries, ‘agglomerate’ means a collection of weakly bound particles or aggregates where the resulting external surface area is similar to the sum of the surface areas of the individual components, ‘aggregate’ means a particle comprising strongly bound or fused particles;

*“Nanomaterial’ means a natural, incidental or manufactured material consisting of solid particles that are present, either on their own or as identifiable constituent particles in aggregates or agglomerates, and where 50% or more of these particles in the number-based size distribution fulfil at least one of the following conditions:*

- *one or more external dimensions of the particle are in the size range 1 nm to 100 nm;*
- *the particle has an elongated shape, such as a rod, fibre or tube, where two external dimensions are smaller than 1 nm and the other dimension is larger than 100 nm;*
- *the particle has a plate-like shape, where one external dimension is smaller than 1 nm and the other dimensions are larger than 100 nm.*

*In the determination of the particle number-based size distribution, particles with at least two orthogonal external dimensions larger than 100 μm need not be considered.*

*However, a material with a specific surface area by volume of < 6 m^2^ /cm^3^ shall not be considered a nanomaterial’.*

In contrast, the USA FDA has not established a regulatory definition for terms such as nanotechnology, nanomaterial, or related terms. Instead, it provides a series of considerations in its Guidance for Industry (*Considering Whether an FDA-Regulated Product Involves the Application of Nanotechnology*) to help manufacturers determine if their product involves nanotechnology (60).

*(1) whether a material or end product is engineered to have at least one external dimension, or an internal or surface structure, in the nanoscale range (approximately 1 nm to 100 nm).*

*In addition, because materials or end products can also exhibit related properties or phenomena attributable to a dimension(s) outside the nanoscale range of approximately 1 nm to 100 nm that are relevant to evaluations of safety, effectiveness, performance, quality, public health impact, or regulatory status of products, we will also ask:*

*(2) whether a material or end product is engineered to exhibit properties or phenomena, including physical or chemical properties or biological effects, that are attributable to its dimension(s), even if these dimensions fall outside the nanoscale range, up to one micrometer (1,000 nm).*

Globally applicable definitions are provided by ISO standard 80,004–1:2023 *Nanotechnologies – Vocabulary Part 1: Core vocabulary*:

- *Nanoscale: length range approximately from 1 nm to 100 nm.*
- *Nanomaterial: material with any external dimension in the nanoscale (3.1.1) or having internal structure or surface structure in the nanoscale.*

Despite the broad consensus on the significance of nanoscale materials, regulatory agencies offer varying specific definitions. For example, the US Environmental Protection Agency and novel food regulations do not establish a minimum size threshold, while the FDA’s guidance allows considering particles up to 1.000 nanometers. The EU’s recommendation, along with chemical and biocide regulations, requires that at least 50% of a material’s particles be within the 1–100 nanometer range.

Consequently, whether a substance is classified as a nanomaterial can hinge on its regulatory context and geographic region, with subtle differences applied by each governing body (61, 62). Nonetheless, definitions based on size thresholds must be treated with caution, as the dimensions of nanomaterials can be influenced by their surrounding medium (e.g., through aggregation, agglomeration, or surface modifications). Whichever definition is employed, it remains essential that all substances and materials receive the requisite safety assessments for their intended applications. Furthermore, not all nanomaterials exhibit novel properties driven by size, while certain emerging attributes (such as color changes observed at the nano-scale) have yet to be correlated with any known or plausible hazard or risk.

### 4.2 Physicochemical characterization

Nanomedicine has witnessed substantial growth, leading to an increased focus on the regulatory aspects of NHP characterization. Authorities such as the EMA or the FDA underscore the importance of physicochemical parameters in assessing the quality, efficacy, and safety of NHPs. These minimal parameters include particle size distribution, chemical composition, drug loading, and release kinetics (63).

Despite the necessity for in-depth characterization, the current standardized methods can be limited and often inadequate for innovative nanoformulations. This has created a need for the harmonization of protocols and methodologies to ensure regulatory approval and compliance. The harmonization process is crucial for advancing nanomedicine and protecting public health (63, 64).

A foundational step in evaluating NHPs for therapeutic use is rigorous physicochemical characterization, which is vital for ensuring the product’s quality, safety, and efficacy. Techniques like dynamic light scattering (DLS) and electron microscopy (EM) are commonly used but have limitations and are often paired with other methods for a more accurate assessment. Agencies recommend validated and standardized methods, with guidance from organizations such as ISO and ASTM International (63).

Initiatives like the ‘Assay Cascade Protocols’ support the development of reliable nanoparticle characterization techniques (65). Such initiatives provide structured approaches based on effective methods, aiding both industry and academia in regulatory compliance for nanoparticle-based products. Institutions like the Nanotechnology Characterisation Laboratory (NCI-NCL in the USA and EU-NCL in Europe) and the OECD’s Working Party on Manufactured Nanomaterials (WPMN) play an instrumental role as well (66).

### 4.3 Biocompatibility, nanotoxicity, pharmacokinetics and pharmacodynamics

The physicochemical characteristics of *in vivo* application of NHPs are linked to their biological behavior. Subtle variations in these characteristics can result in significant changes in their mode of action and toxicity (67). When NHPs interact with biological media, they adsorb medium constituents onto their surface, creating a protein-rich corona, which transforms the nanoparticles into a hybrid system. This significantly alters their pharmacokinetics compared to small drug molecules, presenting a challenge in understanding their safety profile and meeting regulatory expectations (29, 57, 68–72, 100).

Nanotoxicology, which studies the adverse effects of nanomaterials, reveals that NHPs can cause toxicity through mechanisms such as oxidative stress, leading to cellular damage, inflammation, and metabolic dysfunctions (73). These can result in cellular dysfunction and death mechanisms beyond oxidative stress, such as apoptosis, necrosis, and autophagy (58, 74). Additionally, nanoparticles can disrupt molecular and biochemical pathways, leading to mitochondrial damage and further cellular stress, as well as induce cell cycle arrest and epigenetic changes that affect cell division and repair processes, contributing to cytotoxicity (58, 74).

In response to these challenges, organizations such as the NCL have been instrumental in developing new methods and protocols. The NCL has assembled extensive databases and standardized analytical protocols that are invaluable for addressing questions raised by regulatory bodies and guiding the development of NHPs. These resources support the advancement of nanomedicine by refining methodologies and establishing safety and efficacy relationships (56).

Regulatory authorities assess NHPs on a case-by-case basis, often adapting safety and toxicity assessment strategies from conventional health products. The safety evaluation of NHPs encompasses a wide spectrum, from molecular characterization to clinical predictability. Regulatory agencies must account for the multicomponent nature of these products. Embracing safe-by-design principles, employing advanced biological models, and promoting collaborations between academia and industry are vital trends for overcoming challenges and ensuring the safe and effective clinical application of NHPs (56).

### 4.4 Scale-up and manufacturing of NHPs

The scale-up and manufacturing phases introduce distinctive challenges in the rapidly advancing sector of nanomedicine. An in-depth understanding of the interacting components is necessary to identify the product’s essential characteristics, which is crucial for determining critical manufacturing steps and analytical benchmarks to ensure product reproducibility (75–77).

Nanoparticle production methodologies are broadly categorized into ‘top-down’ and ‘bottom-up’ strategies. The top-down approach involves reducing larger entities into smaller particles, while the bottom-up strategy involves assembling smaller components into more sophisticated structures. The formulation process may include techniques such as homogenization, sonication, milling, emulsification, crosslinking, and lyophilization (78).

The Quality by Design (QbD) approach addresses these complexities effectively by defining critical quality attributes (CQAs) for a quality target product profile (QTPP) early in the development process. It advocates a systematic and risk assessment-based strategy for controlling the development and the manufacturing process (ICH Q8 (R2), ICH Q9, ICH Q10). By mapping and parameterising variables that significantly impact the safety and efficacy profile of the final product, QbD facilitates reproducibility, batch-to-batch consistency, and scaling up, increasing the likelihood of regulatory approval for complex products (79, 80).

Identifying key process conditions that affect the desired attributes and functionality of the product is essential. These conditions include the ratios of polymers, drugs, targeting moieties, the type of solvents and emulsifiers used, as well as mixing parameters, temperature, pressure, ionic strength, and pH. Such factors can profoundly influence the chemical structure and purity of the active components, especially impacting the biological effectiveness of macromolecules (81, 82).

For instance, the production of liposomes demonstrates the importance of manufacturing process control. Different preparation methods result in various vesicle structures, each with specific stability concerns (83). The FDA emphasizes the need for tight manufacturing control due to the susceptibility of liposome drug products to alterations in manufacturing conditions, including scale, shear force, and temperature (84).

Sterility presents another substantial challenge, particularly for nanomedicines requiring sterile administration routes. The particle size and structure significantly influence the feasibility of sterilisation methods, with filtration posing challenges for rigid nanoparticles near the nominal pore size of standard filtration membranes (85).

Environmental safety concerns also play a vital role in nanoparticle manufacturing (86, 87). Special care must be taken when handling dry nanomaterials to prevent aerosolization and subsequent pulmonary toxicity risks. The potential for dermal exposure needs adequate protection for personnel involved in manufacturing (88, 89).

In summary, the scale-up and manufacturing of NHPs present various regulatory challenges that require strict process management and a commitment to safety practices to ensure the consistent delivery of safe and efficacious nanomedicines. The implementation of a QbD approach is essential to navigate the complexities of these products and to enhance the probability of regulatory success.

## 5 Conclusion

In conclusion, the evolving regulatory landscape for NHPs across the EU and the US—and increasingly in emerging markets such as China and Japan—continues to face persistent hurdles. These include the absence of harmonized definitions, complex physicochemical characterization requirements, and intricacies in evaluating nanotoxicity. Existing frameworks often extend from established pharmaceutical or medical device regulations, contributing to prolonged approval timelines and restricted patient access.

A prominent challenge lies in how follow-on nanomedicines (nanosimilars) navigate existing approval pathways. In the EU, “hybrid” applications balance varying degrees of preclinical and clinical data, yet the absence of nanotechnology-specific guidelines often complicates or prolongs these submissions. Meanwhile, in the US, the ANDA pathway does not seamlessly address nanosimilars, creating uncertainties around equivalence criteria and, in turn, timeline predictability. These gaps underscore the need for clear regulatory guidance on equivalence standards for complex nanostructures.

Nevertheless, several initiatives at national and international levels are streamlining nanoparticle characterization and safety assessment. For instance, programs such as the ‘Assay Cascade Protocols’ (65) and the Nanotechnology Characterisation Laboratory (NCI-NCL in the USA and EU-NCL in Europe) provide structured approaches for evaluating nanoscale materials used in health products. These endeavors reduce uncertainties by promoting uniform data reporting and robust methodologies, thereby fostering global collaboration and more predictable pathways for NHP authorization.

Additionally, emerging thematic areas are shaping the future of nanomedicine regulation. Artificial Intelligence (AI) is now recognized as a powerful tool to expedite NHP characterization and toxicity assessment. AI-driven algorithms can process large volumes of nanotoxicology data to identify critical parameters—such as size, shape, and surface charge—that influence safety and efficacy. These insights can facilitate “safer-by-design” principles, ultimately accelerating product development while minimizing the need for extensive animal testing. However, AI-based findings often lack transparency from a regulatory standpoint, necessitating improved model interpretability for healthcare applications. Recent advances in causal and graph neural networks show promise in elucidating cause-effect relationships, thereby enhancing the reliability and acceptance of AI-supported decision-making in nanomedicine (90).

Environmental considerations associated with NHP development are also gaining momentum. Pharmaceutical residues have been detected in different ecological compartments (86, 91), and the hazards posed by nanomanufacturing by-products cannot be overlooked (88). Green nanotechnology presents an opportunity to minimise these risks (92, 93). Regulatory bodies, including the European Environment Agency (EEA), already mandate environmental risk assessments for new medicinal products (98). Likewise, requirements for safe disposal of devices and waste are embedded in regulations for medical devices. In practical terms, NHPs are commonly manufactured using either top-down methods—fragmenting larger materials into nanoscale structures, which tends to generate more waste (94, 95)—or bottom-up processes that assemble nanoparticles from atomic or molecular species (95). The bottom-up approach can also incorporate biological reactions (96, 97), thus aligning with ‘green nanomanufacturing’ principles such as reduced toxicity, biodegradability, and energy efficiency [(CHMP), 2006]. Incorporating nanomanufacturing criteria into a classification system would enable stakeholders to quickly identify products whose environmental impact needs special attention, guiding both the development cycle and regulatory scrutiny.

Altogether, efforts to modernise regulatory frameworks and encourage standardized testing, coupled with the emergence of AI-driven methodologies and the shift toward greener nanomanufacturing, signal a promising future for nanomedicine. Yet further collaboration—across scientific, governmental, and industrial spheres—is essential to fully harness these opportunities. By advancing sustainable production methods, refining safety assessment with AI, and harmonizing data requirements internationally, the global community can better balance innovation with public health, ultimately ensuring that nanotechnology-enabled health products reach patients both safely and efficiently.
